# A rare presentation of Erdheim Chester disease in a pediatric patient subsequently cured on the LCH III protocol

**DOI:** 10.1002/cnr2.1304

**Published:** 2020-10-06

**Authors:** Aditya Kumar Gupta, Abdul Wajid M, Jagdish P. Meena, Sreedharan T. ArunRaj, Asit Mridha, Priyanka Naranje, Rakesh Kumar, Rachna Seth

**Affiliations:** ^1^ Division of Pediatric Oncology, Department of Pediatrics All India Institute of Medical Sciences New Delhi India; ^2^ Department of Nuclear Medicine All India Institute of Medical Sciences New Delhi India; ^3^ Department of Pathology All India Institute of Medical Sciences New Delhi India; ^4^ Department of Radiodiagnosis All India Institute of Medical Sciences New Delhi India

**Keywords:** Erdheim Chester disease, LCH‐III protocol, pediatrics

## Abstract

**Background:**

Erdheim Chester disease (ECD) is very rare in pediatrics with no standard treatment guidelines. Here we present the case of a pediatric ECD patient who was cured with a Langerhan cell histiocytosis (LCH) directed chemotherapy protocol.

**Aim:**

The aim of the report was to publish this rare presentation of ECD in pediatrics and highlight the complete response obtained to treatment.

**Methods:**

The details of the patient were extracted by a retrospective review of her clinical records.

**Results (Case):**

An 11 years old girl presented with fever and bone pain. On investigating she had multiple lytic bony lesions scattered throughout her skeleton. A biopsy from one of the bone lesions confirmed the diagnosis to be ECD. ECD is very rare in pediatrics and this case adds to the existing list of 11 previously reported ones. Also, worth mention is the fact that the child presented with isolated skeletal involvement in form of multiple osteolytic lesions. The child was started on the LCH‐III protocol on which she achieved a cure.

**Conclusion:**

Lytic bone lesions in a child may be present in ECD. A subset of ECD may have good response to LCH like chemotherapy.

## 1 INTRODUCTION

Erdheim Chester disease (ECD) is non Langerhan cell histiocytosis (LCH), which is very rare in pediatrics. The name of the disease comes from Jacob Erdheim and William Chester, who initially described it in 1939.^1^ In adults the disease presents with a triad of bone pain, proptosis and diabetes insipidus. Most pediatric patients present with bone involvement and the radiology typically shows osteosclerotic lesions. The *BRAF* mutation is found in up to 50% of patients and the histopathology typically reveals foamy lipid laden histiocytes that are CD68 positive and negative for CD1a and S‐100 protein.^2^ There is no standard treatment for ECD in pediatrics and the prognosis is poor with a mean survival of about 32 months.^3^ We report the case of an 11 years old girl who was diagnosed with ECD and she achieved a complete response on a LCH directed protocol.^4^

## 2 CASE REPORT

An 11 years old girl presented to our hospital with a history of intermittent fever for 5 months. The fever was high grade and was relieved temporarily by antipyretics. The fever used to occur irregularly with intermittent periods of defeverescence. She had been treated with multiple courses of antibiotics without relief. Her chest x ray (CXR) had revealed lytic lesions in her ribs after which she had been referred to our centre.

Upon presentation to our hospital she had been afebrile, but had bone pain at multiple sites. There were no other complains. On examination, she had tenderness on deep palpation of the long bones. The systemic examination was otherwise unremarkable. Her sexual maturity rating was appropriate for age.

**FIGURE 1:**
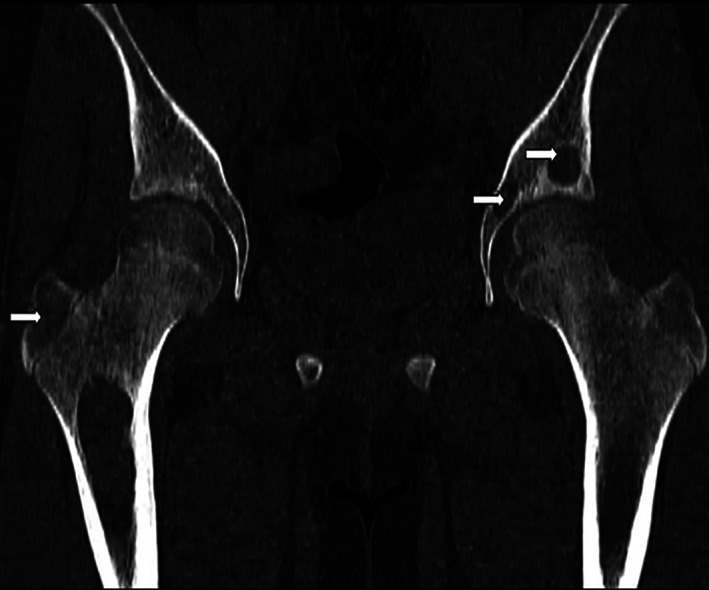
Coronal computed tomography image in bone window shows multiple geographical lytic lesions in bones in right femur and left pelvic bones (white arrows)

**FIGURE 2:**
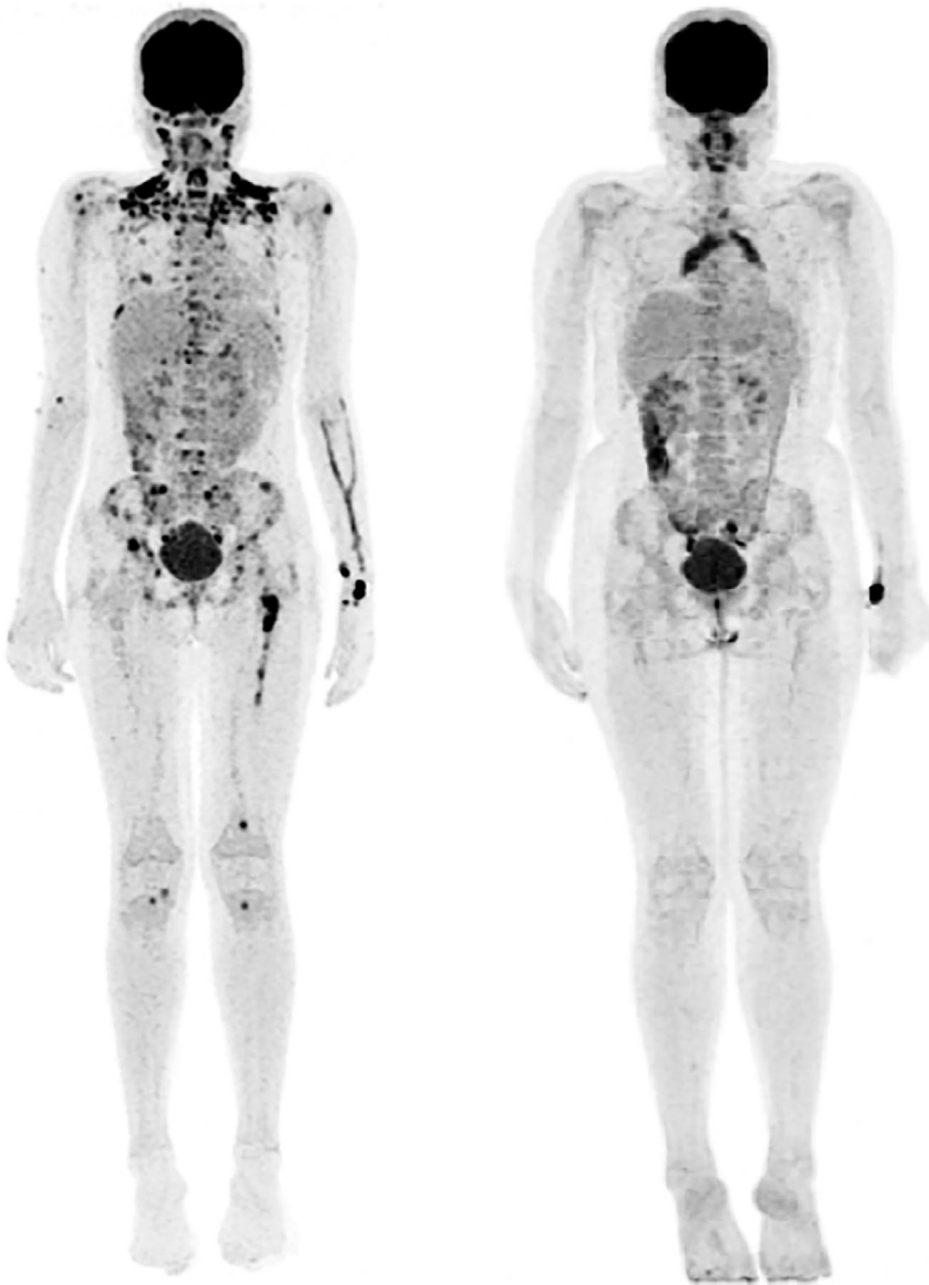
Positron emission tomography (PET) scan image showing FDG avid lesions involving multiple bones at diagnosis (left panel). The PET scan image after completion of treatment (right panel) shows a complete response

Her complete blood count, liver function tests and renal function tests were normal. The urine investigations were unremarkable. The CXR showed a normal lung parenchyma and lytic lesions in the ribs, clavicle, sternum and scapulae. The Mantoux test was negative. A skeletal survey by X‐ray and computed tomography (CT) scan revealed lytic lesions in multiple bones (Figure 1). The positron emission tomography (PET) scan revealed multiple fluorodeoxyglucose (FDG) avid lesions throughout her skeleton (Figure 2).

**FIGURE 3:**
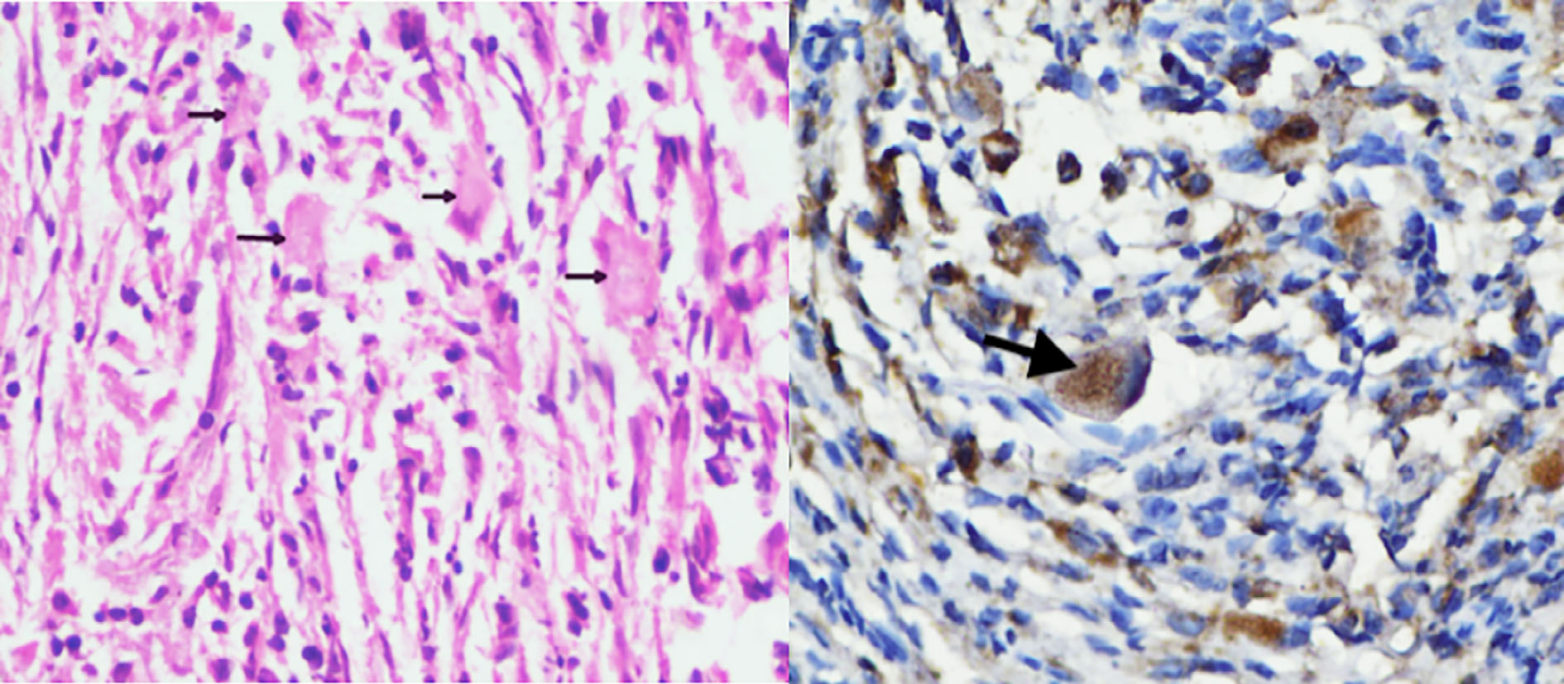
Hematoxylin and Eosin stained sections (x400) of the bone biopsy showing foamy histiocytic infiltrate on the left panel (black arrows) with pale eosinophilic abundant cytoplasm in a background of fibroblastic proliferation and lymphocytic infiltrate. The histiocytic cells were CD1a and S‐100 negative but showed positivity for CD 68 (black arrow on the right panel)

A CT guided biopsy was done from one of the FDG avid bone lesions that revealed a foamy histiocytic infiltrate with pale eosinophilic abundant cytoplasm in a background of fibroblastic proliferation, and lymphocytic infiltrate. The histiocytic cells were CD1a and S‐100 negative but showed positivity for CD68 (Figure 3). A diagnosis of ECD was made.

There is no standard treatment for ECD. Previous pediatric patients have been treated with various agents like interferon‐α, vinca alkaloids, steroids, anthracyclines and radiotherapy. After taking into consideration the feasibility and financial status of the family the child was given a trial on the LCH III protocol^4^ (Multifocal bone disease; Group 3).

The child achieved partial remission after the first phase of treatment consisting of prednisolone and weekly vinblastine (initial treatment course‐1). Subsequently a second cycle was administered (initial treatment course‐2) following which a complete response was obtained and the child was then put on a 12 months continuation treatment consisting of daily oral 6‐mercaptopurine and three weekly vinblastine and prednisolone. The PET scan post completion of treatment showed complete remission of the disease (Figure 2). The child is now 12 months post completion of treatment and continues to be in sustained remission.

## 3 DISCUSSION

ECD is a very rare disease in pediatrics. Only 11 cases have been reported in the literature in the pediatric age group.^3^The exact etiology of the disease is not clear but a mutation in the *BRAF* gene can be found in approximately half of the patients.^2^ The clinical triad of ECD comprises of bony pain, bilateral painless exophthalmos and diabetes insipidus. The bone involvement in ECD typically consists of sclerotic symmetrical metaphyseal lesions in the long bones. Organ involvement could consist of diabetes insipidus, xanthelasma, interstitial lung disease, bilateral adrenal enlargement, retroperitoneal and perirenal fibrosis, ureteral stenosis, renal impairment, testis infiltration, and central nervous system and cardiovascular involvement.^5^

On histopathological examination, the histiocytes in ECD are non‐Langerhan's foamy histiocytes, which lack the Birbeck granules and nuclear grooves that are typical of LCH. They demonstrate positivity for CD68 and CD163, and are negative for the S‐100 protein and CD1a. This helps in differentiating ECD from LCH, in which histiocytes are positive for CD1a and S100.^6^

Interferon‐*α*, is the first‐line drug in the symptomatic ECD in adults but the optimal dose and duration is not defined. Currently there are no standard treatment guidelines for pediatric ECD. Steroids with or without chemotherapy, radiotherapy and hematopoietic transplant have been tried with variable success. *BRAF* mutations have been detected in some cases of ECD and future options are being explored with *BRAF* inhibition using Vemurafenib.^7^

Our case report adds to the few available in literature where a pediatric patient was diagnosed with ECD. ECD involving only the bones is very rare. Whereas the typical lesions described in ECD are osteosclerotic lesions, the above patient had lytic lesions on her imaging. In the series of 11 previously known pediatric ECD patients collated by Khan et al,^3^ six patients had osteosclerotic lesions, three patients had a mixture of osteolytic and osteosclerotic lesions whereas two had pure osteolytic lesions.

The child responded to the conventional chemotherapy known to be effective in LCH. The patient is now 12 months post treatment completion and continues to be in good health. Previous pediatric patients have been treated on either steroids or interferon‐*α*, but the prognosis has been poor. This patient is the first case reported where a successful response to a LCH directed treatment was demonstrated in ECD. As had been shown in more recent studies LCH and ECD are both inflammatory myeloid neoplasms caused by mutations in the MEK‐extracellular signal‐regulated kinase (ERK) signalling pathway, most commonly involving the *BRAF* gene.^8^ This can explain the response of ECD to the chemotherapy regimen that has been used for its commoner counterpart that is, LCH. Whether it is only a subset of ECD that would respond to this treatment needs further investigation.

## CONFLICT OF INTEREST

The authors declare no potential conflict of interest.

## AUTHORS CONTRIBUTIONS

All authors had full access to the data in the study and take responsibility for the integrity of the data and the accuracy of the data analysis. *Conceptualization*, A.K.G.; *Data curation*, A.K.G., A.W.M., S.T.A., A.M., P.N., R.K., R.S.; *Investigation*, A.K.G., A.W.M., J.P.M., S.T.A., A.M., P.N., R.K., R.S.; *Resources*, A.M., P.N., R.K., R.S.; *Writing ‐ Original Draft*, A.K.G., A.W.M.; *Writing ‐ Review & Editing*, A.K.G., A.W.M., J.P.M., S.T.A., A.M.,P.N., R.K., R.S.; *Visualization*, A.K.G., J.P.M., R.S.; *Supervision*, A.K.G., R.S.

## ETHICAL STATEMENT

The informed and written consent to publish this case report was obtained from the patient's father and the publication was done in accordance to the institute ethics committee guidelines.

## Data Availability

The data that support the findings of this study are available on request from the corresponding author. The data are not available publicly due to privacy or ethical restrictions.

## References

[1] Veyssier‐Belot C , Cacoub P , Caparros‐Lefebvre D , et al. Erdheim‐ Chester disease: clinical and radiologic characteristics of 59 cases. Med Baltim. 1996;75(3):157e69. 10.1097/00005792-199605000-00005 8965684

[2] Vallonthaiel AG , Mridha AR , Gamanagatti S , et al. Unusual presentation of Erdheim‐Chester disease in a child with acute lymphoblastic leukemia. World J Radiol. 2016;8(8):757e63. 27648170 10.4329/wjr.v8.i8.757 PMC5002507

[3] Khan MR , Ashraf MS , Belgaumi AF . Erdheim Chester disease. An unusual presentation of a rare histiocytic disease in a 3‐year old boy. Pediatr Hematol Oncol J. 2017;2:59‐62.

[4] Gadner H , Minkov M , Grois N , et al. Therapy prolongation improves outcome in multisystem Langerhans cell histiocytosis. Blood. 2013;121(25):5006‐5014. 23589673 10.1182/blood-2012-09-455774

[5] Diamond EL , Dagna L , Hyman DM , et al. Consensus guidelines for the diagnosis and clinical management of Erdheim‐ Chester disease. Blood. 2014;124(4):483e92. 24850756 10.1182/blood-2014-03-561381 PMC4110656

[6] Clerico A , Ragni G , Cappelli C , Schiavetti A , Gonfiantini M , Uccini S . Erdheim‐ Chester disease in a child. Med Pediatr Oncol. 2003;41(6):575e7. 14595723 10.1002/mpo.10402

[7] Campochiaro C , Tomelleri A , Cavalli G , Berti A , Dagna L . Erdheim‐Chester disease. Eur J Intern Med. 2015;26(4):223e9. 25865950 10.1016/j.ejim.2015.03.004

[8] Milne P , Bigley V , Bacon CM , et al. Hematopoietic origin of Langerhans cell histiocytosis and Erdheim‐Chester disease in adults. Blood. 2017;130(2):167‐175. 28512190 10.1182/blood-2016-12-757823 PMC5524529

